# Locus-of-Hope Intervention in School: A Localized Strength-Based Mental Health Promotion Program

**DOI:** 10.3389/ijph.2024.1607010

**Published:** 2024-02-21

**Authors:** Justin Vianey Embalsado

**Affiliations:** ^1^ Department of Psychology, College of Arts and Sciences, Angeles University Foundation, Angeles, Philippines; ^2^ Health Sciences Program, Graduate School, Angeles University Foundation, Angeles, Philippines; ^3^ Psychology Program, Graduate School, Angeles University Foundation, Angeles, Philippines

**Keywords:** positive education, hope intervention, locus of hope, strength-based mental health, Filipino student

**The IJPH series “Young Researcher Editorial” is a training project of the Swiss School of Public Health.**

The Philippine Universal Healthcare Act [1] aims to promote complete health to the Filipino people. The Health Promotion Framework System was based on the UHC to cater to people-oriented interventions to maintain positive physical and mental health. UHC designated schools as healthy communities to promote complete health (e.g., physical and mental) [2]. Developing mental health programs based on the student’s psychological capacities can help mitigate adverse mental health and manage the demand for specialized services. The programs can address the lack of professionals, facilities, and government funding for specialized mental health services [3]. WHO [4] contends that mental health is not limited to deficits and weaknesses but also about strengths and positive mental health. The emphasis on psychological strengths involves the promotion of positive mental health by developing psychological capacities (adaptive coping - problem-solving, cognitive reappraisal) and resources (i.e., hope) [4, 5]. However, there is a lack of evidence on the efficacy of hope intervention in schools. Research on hope and adaptive coping mainly focuses on surveys and interviews. Even though there are recommendations on hope interventions, there is still limited research in this area. Hope interventions were conducted in Western societies in the United States, Australia, and Portugal, while research in Eastern societies was only in Hong Kong and Singapore [6, 7].

Developing and assessing strength-based research is essential in promoting positive mental health in schools [3]. The schools should inform, educate, and empower students by developing mental health policies and programs. According to the hope model, students can reach their goals by developing agency and pathway thinking [5, 8]. Agency refers to the student’s belief in themselves, while pathway thinking refers to strategies and alternatives to reach their goals [5]. A student’s goal can be about their academic performance or mental health. Thus, hope aims to develop psychological capacities by guiding them to believe in themselves and develop ways to reach their goals. Hope differs from other psychological resources like self-efficacy, optimism, and resilience since it includes solutions for solving problems rather than focusing on their belief in themselves (i.e., self-efficacy), and it is not limited to a positive outlook in adverse situations (i.e., optimism), or sole emphasis on recovering and bouncing back from difficulties (i.e., resilience) [9]. Hope activates even without challenging events, hope prepares an individual to face challenges by believing in oneself and developing strategies to attain their goals. Hope is predictive of adaptive coping mechanisms like positive reframing, where an individual develops a positive framework in life when facing adverse events.

Applying hope in context, the hope model focused on individualistic psychological resources. Cultures and communities emphasize relationships and harmony with others and source their hopeful thinking from others. Bernardo [10] extends the hope theory by identifying the external locus of hope (i.e., parents, peers, and spirituality). The Philippines, a collectivist society, tends to source psychological resources from others. Specifically, the locus of hope accounts for internal and external locus, including parents, peers, and spirituality. Filipinos may source their belief from themselves with the support of the aforementioned social agencies and adhere to their guidance in developing strategies to reach their goals in life. The inclusion of social agency extends the hope model and underscores the role of culture and community in honing psychological resources. In schools, teachers serve as agents of hope. There is currently no evidence of teachers as a locus of hope, but conceptualizations of mental health intervention suggest including local or indigenous agents of psychological sources. In schools, teachers are social agents who mold students to achieve their goals; teachers provide students with strategies to achieve their goals and motivate students to believe in their abilities [8]. Teachers may guide students to pass their courses or do better in life [5].

Hope intervention is not limited to overcoming one’s weaknesses; it can help to develop strengths to achieve one’s potential. A randomized controlled trial was conducted in Singapore to measure the impact of hope intervention (e.g., using Snyder’s hope model) on mental health literacy, psychological wellbeing, and stress. The hope intervention group showed increased mental health literacy but failed to improve psychological wellbeing [7]. To make hope intervention more attuned to collectivist culture, Bernardo and Sit [6] recommend including social agents in implementing the intervention. For instance, rather than focusing on how an individual can achieve their goals, they can be motivated to reflect on how their parents, peers, and spirituality guide them with their goals. Teachers also serve as agents of hope since they help students identify their goals, motivate them to believe in their abilities, and provide guidance to help them reach their goals.
